# User Dynamics and Thematic Exploration in r/Depression During the COVID-19 Pandemic: Insights From Overlapping r/SuicideWatch Users

**DOI:** 10.2196/53968

**Published:** 2024-05-20

**Authors:** Jianfeng Zhu, Ruoming Jin, Deric R Kenne, NhatHai Phan, Wei-Shinn Ku

**Affiliations:** 1 Department of Computer Science Kent State University Kent, OH United States; 2 Center for Public Policy and Health College of Public Health Kent State University Kent, OH United States; 3 Department of Data Science New Jersey Institute of Technology Newark, NJ United States; 4 Department of Computer Science and Software Engineering Auburn University Auburn, AL United States

**Keywords:** reddit, natural language processing, NLP, suicidal ideation, SI, online communities, depression symptoms, COVID-19 pandemic, bidirectional encoder representations from transformers, BERT, r/SuicideWatch, r/Depression

## Abstract

**Background:**

In 2023, the United States experienced its highest- recorded number of suicides, exceeding 50,000 deaths. In the realm of psychiatric disorders, major depressive disorder stands out as the most common issue, affecting 15% to 17% of the population and carrying a notable suicide risk of approximately 15%. However, not everyone with depression has suicidal thoughts. While “suicidal depression” is not a clinical diagnosis, it may be observed in daily life, emphasizing the need for awareness.

**Objective:**

This study aims to examine the dynamics, emotional tones, and topics discussed in posts within the r/Depression subreddit, with a specific focus on users who had also engaged in the r/SuicideWatch community. The objective was to use natural language processing techniques and models to better understand the complexities of depression among users with potential suicide ideation, with the goal of improving intervention and prevention strategies for suicide.

**Methods:**

Archived posts were extracted from the r/Depression and r/SuicideWatch Reddit communities in English spanning from 2019 to 2022, resulting in a final data set of over 150,000 posts contributed by approximately 25,000 unique overlapping users. A broad and comprehensive mix of methods was conducted on these posts, including trend and survival analysis, to explore the dynamic of users in the 2 subreddits. The BERT family of models extracted features from data for sentiment and thematic analysis.

**Results:**

On August 16, 2020, the post count in r/SuicideWatch surpassed that of r/Depression. The transition from r/Depression to r/SuicideWatch in 2020 was the shortest, lasting only 26 days. Sadness emerged as the most prevalent emotion among overlapping users in the r/Depression community. In addition, physical activity changes, negative self-view, and suicidal thoughts were identified as the most common depression symptoms, all showing strong positive correlations with the emotion tone of disappointment. Furthermore, the topic “struggles with depression and motivation in school and work” (12%) emerged as the most discussed topic aside from suicidal thoughts, categorizing users based on their inclination toward suicide ideation.

**Conclusions:**

Our study underscores the effectiveness of using natural language processing techniques to explore language markers and patterns associated with mental health challenges in online communities like r/Depression and r/SuicideWatch. These insights offer novel perspectives distinct from previous research. In the future, there will be potential for further refinement and optimization of machine classifications using these techniques, which could lead to more effective intervention and prevention strategies.

## Introduction

### Background

In the United States, approximately 36% of Americans have self-reported experiences of serious loneliness during the COVID-19 pandemic, with an even more concerning 43% having acknowledged an intensified sense of isolation since its inception [1]. This intensified loneliness, compounded by the challenges of the pandemic, has worsened the struggles of individuals already dealing with preexisting mental health issues. Suicidal ideation (SI) is common among people with mental health problems, particularly depression [2], and the combination of pandemic-induced loneliness and existing mental health concerns further escalates the risk.

Identifying individuals at risk of suicide can be challenging due to the overlap between its warning signs and the symptoms of depression. Key indicators include significant changes in eating and sleep patterns; loss of interest in work, school, and community; social withdrawal; reckless behaviors; substance abuse; neglect of personal appearance; and an obsession with death. These challenging circumstances have created a fertile ground for a distressing increase in substance abuse; traumatic stressors; and, most alarmingly, suicidal thoughts [3]. However, detecting and measuring these thoughts is not easy, as thoughts of death, dying, or suicidal plans are often not expressed directly. The challenge is further amplified by the fact that such thoughts can change in intensity and frequency [4]. Clinicians are now requested to provide an in-depth investigation into the mind of a person who is suicidal, an assessment adjunctive to the psychiatric evaluation [5].

A closer examination reveals that working with individuals at risk of suicide reveals a complexity that defies the constraints of the outdated medical model. Warning signs often precede suicidal thoughts and actions, and individuals with suicidal tendencies frequently grapple with mental anguish as a common thread woven through various adverse life events [6]. In contrast, the widespread use of social media platforms offers a new way for people to express their emotions continuously. This digital space has the potential to better understand people’s mental health. Importantly, using social media platforms allows researchers to study larger groups, making their findings stronger and more useful.

Our main goal is to gain insights into early signs of distress by analyzing social media content, focusing on transition patterns, emotion tones, and themes associated with suicidal depression. We achieve this through the application of natural language processing (NLP) and statistical methodology. By comprehending the multifaceted perspectives, coupled with appropriate interventions, depression can be treated and suicide can be prevented.

### Prior Research

The emergence of online communities, largely facilitated by platforms like social media, has carved out a space for people to connect, share experiences, and support each other [3]. This surge in online activity has also empowered the social NLP research community, allowing for advancements in understanding mental health indicators from self-reported text [7].

Research in this area has focused on extracting features and patterns from social media data to identify potential risks, and various behavioral features such as the number of posts, the rate of retweets [8], the frequency of absolutist word [9], and the prevalence of emotional characteristics [10,11]. In addition, studies have explored the transition of users to subreddits like r/SuicideWatch [11], harnessing social media data in identifying mental health symptoms [12,13]. Meanwhile, another line of investigation focuses on detecting and predicting different mental health categories. Sarkar et al [12] explored the prediction of depression and anxiety on Reddit using a multitask learning approach that combines word embedding features, pretrained BERT models, and topic modeling features (ie, latent Dirichlet allocation and BERTopic). In a parallel, Kayalvizhi and Thenmozhi [13] established a gold standard data set for identifying levels of depression, such as not depressed, moderately depressed, and severely depressed from social media posts . In addition, Ghosal and Jain [14] proposed a novel framework to differentiate between depression and suicidal risk content using fastText embedding for contextual analysis, Term Frequency–Inverse Document Frequency vector for the relevance of terms, and machine learning classifier XGBoost for accurate classification. More Recently, Cai et al [15] leveraged depressive symptom score calculation to obtain the cosine similarity between posts and depressive symptom descriptions and then predict the presence of depression.

NLP has demonstrated strong performance in tasks such as text classification and sentiment mining. Tadesse et al [16] effectively used NLP techniques to identify specific terms indicating depression, emphasizing the potential of NLP in uncovering mental health indicators. In addition, Zhang et al [17] used NLP to characterize changes in mental health support groups on Reddit during the pandemic. The use of BERT-based models has shown promise in detecting mental health illnesses [17]. Kim et al [18] used BERT to extract textual features from mental health subreddits, analyzing linguistic characteristics. Demszky et al [19] used BERT in transfer learning experiments to assess emotional content on Reddit. Furthermore, Ramirez-Cifuentes et al [20] developed predictive models for substance abuse and mental health issues using enhanced representations with statistical and deep learning approaches on social media posts. Baird et al [21] and Alhaj et al [22] used BERTopic to analyze mental health texts on social media platform, enhancing classification accuracy.

However, existing studies [8-19,21-25] have several challenges, including limited data set sizes and time frames and a focus on specific mental health issues without considering the overlap between them. To address these challenges, our study focused on posts from overlapping users in r/Depression and r/SuicideWatch, using trend analysis and survival analysis, emotion feature identification, and machine learning classification to gain insights into posting behavior, emotional expressions, and prevalent topics associated with individuals who may be struggling with suicidal depression.

Our research aims to answer the following research questions (RQs): (RQ1) What are the patterns and trends in user engagement behaviors across both r/Depression and r/SuicideWatch subreddits over time? (RQ2) How do users transition between participating in r/Depression to engaging with r/SuicideWatch subreddits? (RQ3) What emotional expressions and signs of depression are prevalent among overlapping users in r/Depression? (RQ4) What topics are discussed by overlapping users in the r/Depression, categorized based on SI labels?

## Methods

### Data Collection

In our research, we used Reddit, a widely used online platform, to collect data from 2 mental health–related subreddits: r/Depression and r/SuicideWatch. To compile our data set, we accessed the archives of Reddit submission [24], covering the time span from 2019 to 2022. During this data collection period, we gathered around half a million posts. Specifically, we collected over 285,000 posts from >165,000 unique users in the r/SuicideWatch subreddit. For the r/Depression subreddit, our data set included >444,000 posts contributed by over 248,000 distinct users.

While we do not possess detailed demographic information about users within these specific subreddits, we do have insights into the broader Reddit user base. Reddit boasts an impressive 430 million monthly active users, with 74% of them being men and 25.8% being women. Most users fall within the age range of 18 to 29, and a substantial 63% of the users hold at least a bachelor’s degree [25]. This reflects the quality of discussions and content found on Reddit, as users tend to be well-informed and educated on a variety of topics. This statistic is also important to consider when creating content for Reddit, as it suggests that the audience is likely to be more sophisticated than other social media platforms.

### Data Cleaning

The proper execution of data preprocessing techniques is essential to ensure the accuracy of subsequent data analysis [26]. In this study, we used thematic and topic modeling strategies that generate document embeddings. This process typically eliminates the need for preprocessing by extracting the central theme from the entire document, which is usually sufficient in most cases. However, in instances where the data contain extraneous elements, such as HTML tags, it is prudent to remove them. These tags rarely contribute to the semantic content of a document and can be safely discarded.

Our data cleaning process involved several steps. First, we expanded contractions to enhance textual clarity. Next, we removed nonalphanumeric characters, replacing them with spaces to cleanse the corpus. We standardized all text to lower case to maintain consistency and avoid case-sensitive duplicates. Finally, we replaced empty strings with “not a number” values to indicate missing or inexpressible data points. The not a number designation represents a unique floating-point value used to signify data that are undefined or cannot be represented.

### Exploring Overlapping Users’ Behavior in r/Depression and r/SuicideWatch Subreddits

#### Overview

Exploring the users who participate in both the r/SuicideWatch and r/Depression subreddits provides valuable insights into the common behaviors and emotions within online communities. This analysis deepens our understanding of how individuals interact with these platforms and express thoughts and emotions related to mental health. Using a series of methodologies, this study examined the posts of these overlapping users, covering the period from 2019 to 2022, including the pandemic years. A total of 25,000 individuals who actively posted in both communities were identified.

#### Analysis of Posting Patterns

We conducted a comprehensive investigation into the posting patterns of overlapping users. This analysis involved tracking the weekly number of posts in both communities over time. In addition, we aimed to pinpoint specific times or weeks when users were more active, shedding light on their engagement trends.

#### Analysis of Transition Patterns

To gain insights into the temporal progression of users’ mental health journeys, we used survival analysis. This technique assesses the duration between users’ initial interactions in the r/Depression subreddit and their subsequent engagement in r/SuicideWatch. Survival analysis, a time-to-event methodology, explores the timeline leading to a predefined end point of interest [27]. Analyzing this timeline provides a deeper understanding of individuals’ mental health trajectories within the 2 communities during the COVID-19 pandemic period.

### Analyzing r/Depression Posts by Overlapping Users in r/SuicideWatch

When someone experiences depression with SI as a symptom, it means that they are experiencing suicidal thoughts as part of their overall mental health condition. It is not easy to identify when someone may be thinking about suicide, but changes in behavior, thoughts, or mood could indicate the worsening of their mental health status [28]. In addition, emotions play a fundamental role in human interaction, significantly impacting mental illnesses in terms of behavior, responses, and perspectives. Anger, disgust, sadness, and fear are some negative emotions that people with mental conditions may encounter. Such emotions can be intense and persistent, making it challenging for the individual experiencing them to carry on with their regular activities [29]. The rise and continuance of emotion, their category, and intensity are important clues about suicidal tendencies [30].

With the goal of investigating major depression symptoms and emotional tones within posts from users who overlap between the r/Depression and r/SuicideWatch subreddits, we initially used a pretrained BERT model [31] to encode the associated sentences of major depression symptoms (details provided in Table S1 in Multimedia Appendix 1 [32,33]) and each post. Subsequently, we computed the cosine similarity between them to generate a probability score of each depression symptom in each post, ranging from 0 to 1. In addition, we applied the GoEmotion [19] model to each post to generate the emotion tone features along with their respective scores. Finally, we visualized the matrix relationship of the extracted depression and emotion features within the data set using a heatmap.

### BERTopic Modeling Combined With Fine-Tuned DistilBERT Classification Model

The domain of SI involves contemplating or planning suicide within various contexts and encompasses a spectrum of thoughts. These thoughts range from a general desire to die without any specific method, plan, intent, or action to more active forms of SI that include a specific plan and intent [34].

#### Leveraging the DistilBERT Model

##### Overview

To accurately categorize SI-related content in r/SuicideWatch community, we used the DistilBERT model [32], a more compact general-purpose language representation model. DistilBERT is pretrained and can be fine-tuned to achieve strong performance across various tasks, similar to its larger counterparts. Notably, DistilBERT reduces the BERT model’s size by 40% while retaining 97% of its language understanding capabilities and operating 60% faster. For more information about the model’s explanation, please refer to Multimedia Appendix 1.

Regarding the data, we used the gold standard data set [33], which comprises 500 redditors selected from a pool of 2181 users. Domain experts annotated 448 users using the Columbia-Suicide Severity Rating Scale, with labels encompassing *supportive* (a novel addition to Columbia-Suicide Severity Rating Scale specific to social media), *suicide ideation*, *suicide behavior*, *suicide indicator*, and *suicide attempt*. We transformed the gold standard data set, assigning a label of 1 for instances of SI, while labeling other categories with a value of 0. The sample data set provided in Textbox 1 gives a visual representation of these label assignments.

**Restructured samples from the gold standard data set.**

**Suicidal ideation and posts**

- Value of 0: “It’s not a viable option, and you’ll be leaving your wife behind. You’d Pain her beyond comprehension. It sucks worrying about money, I know that first hand. It can definitely feel hopeless, as you seem to be Tired aware of. Your wife might need to chip in financially. I know time is an issue, but even 10-15 hours a Asthenia could alleviate a lot of the pressure. In the meantime, get your shit together - write that resume tomorrow...” [User 0]
- Value of 1: “I tried to kill myself once and failed badly cause in the moment I wanted to do it I realized that I want to live! I still have suicidal thoughts and I often question myself why I even carry on!” [User 1]

Our approach to enhancing the accuracy of classifying SI in Reddit posts within the r/SuicideWatch community followed a systematic and progressive methodology. The steps described in the following sections outline our approach.

##### Step 1: Data Split and Cross-Validation

We initiated our approach by carefully dividing our data set into 2 essential subsets: the training set and the testing set. Specifically, we allocated 80% of the data for the training set, while the remaining 20% was dedicated to the testing set. This data split allowed us to prevent overfitting and assess our model’s performance more effectively. In addition, we implemented cross-validation techniques to continually evaluate our model’s performance during training.

##### Step 2: Initial Fine-Tuning

We initiated the process by fine-tuning a pretrained model using a designated set of the gold data set. The last layer of the transformer in the DistilBERT model was accessed, and dropout layers were added to prevent overfitting.

##### Step 3: Labeling Raw r/SuicideWatch Posts

Using the refined model from the previous step, we predicted SI labels for 500 posts sourced from the r/SuicideWatch subreddit. These predictions underwent manual validation to ensure their correctness.

##### Step 4: Expansion of Gold Set

The manually validated SI-labeled post obtained from the 500 r/SuicideWatch posts were seamlessly incorporated into the original gold standard set.

##### Step 5: Iterative Fine-Tuning

We executed 3 successive rounds of fine-tuning, using the expanded gold set in each iteration. After each round, we assessed the model’s accuracy on a validation set to measure its performance accurately.

Throughout this iterative process, we maintained a data split ratio of 80% for training and 20% for validation. To optimize accuracy, we used different parameter setting models for training.

#### Unveiling Insights Through BERTopic Model

The methodology used in this study integrates the use of BERTopic [35], a topic modeling technique that leverages sentence embeddings and contextual term frequency–inverse document frequency. This approach results in dense clusters that not only facilitate the generation of interpretable topics but also preserve the essential words with topic representation. BERTopic is supported by 4 key models: embedding, uniform manifold approximation and projection, hierarchical density-based spatial clustering of applications with noise, and CountVectorizer. However, since default parameters may not offer a one-size-fits-all solution, BERTopic allows for the customization of these key models’ parameters to better suit specific use cases (refer to Table S2 in Multimedia Appendix 1).

In addition to the 4 key submodels, we incorporated optional representation tuning into our experiment. We used the following additional models to fine-tune the topic representations: KeyBERT [36], Part Of Speech [37], and OpenAI (OpenAI Inc) [38]. The corresponding OpenAI prompt is as follows: “I have a topic that contains the following documents: [DOCUMENTS] The topic is described by the following keywords: [KEYWORDS]. On the basis of the information above, extract a short but highly descriptive topic label. Make sure it is in the following format: topic: <topic label>.”

Finally, the BERTopic model applied a total of 84,010 posts from overlapping users in the r/Depression subreddit. A SI feature was generated based on users who had posted at least once about SI, identified using the Fine-Tuned DistilBERT Classification Model in the r/SuicideWatch subreddit. Out of these posts, 69,430 posts were labeled with a value of 1 for SI, while the remaining 14,671 posts with a value of 0 within the r/Depression community. Various visualization methods, including visualizing the top term probabilities for each topic and generating a heatmap of the topic similarity matrix, were used to gain deeper insight into topics of the 2 groups.

### Ethical Considerations

In order to protect the privacy and confidentiality of the individuals whose data underwent analysis, a thorough deidentification process was conducted on all study data before analysis. The data used in this study were sourced from publicly available platforms [24] and, as such, do not contain any identifiable information. Furthermore, to enhance data privacy, the sample posts underwent preprocessing, resulting in tokens that make it impossible to discern users’ personal details. Importantly, the data set used in this study is entirely devoid of personal information, such as author names or any other private identifiers. In addition, this research was partially supported by the National Science Foundation project (IIS-2041065), which was approved by the institutional review board at Kent State University under the reference number KSU IRB20-182. The Institutional Review Board at Kent State University was consulted regarding privacy ethics concerns and the study was deemed exempt (KSU 819).

## Results

### RQ1: What Are the Patterns and Trends in User Engagement Behaviors Across Both r/Depression and r/SuicideWatch Subreddits Over Time?

Through the examination of the posting behaviors by overlapping users within these subreddits, we tracked the weekly distribution of their posts over this multiyear period. This thorough approach enabled the identification of patterns and trends that shed light on how these users interacted with both the r/Depression and r/SuicideWatch communities.

Of notable statistical importance are the following findings: the count of unique authors across both communities totaled around 25,000, with an overlapping user ratio of 15% for r/SuicideWatch and 10% for r/Depression. Furthermore, the volume of posts within r/SuicideWatch reached 70,742, with an average post length of 373 (SD 515) words. In contrast, r/Depression featured 84,101 posts, with an average post length of 767 (SD 1017) words. These averages were computed directly from the raw text.

**Figure 1:**
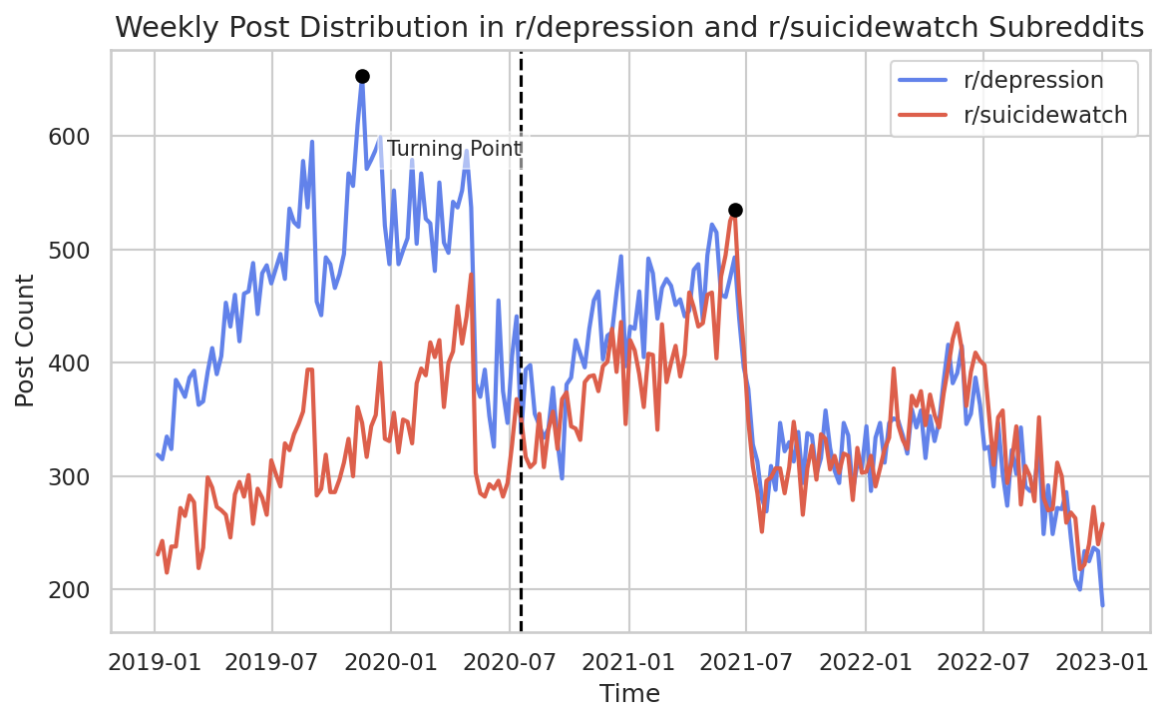
Weekly post distribution in r/Depression and r/SuicideWatch subreddits.

Figure 1 reveals a pivotal turning point on August 16, 2020, when the post count within r/SuicideWatch surpassed that of r/Depression. This critical moment, which closely aligned with the initial spread of COVID-19 in the United States, indicated a notable shift in user engagement. Interestingly, before this turning point, r/Depression had a considerably wider gap in post count compared with r/SuicideWatch. However, after August 2020, the post curves of the 2 subreddits converged. This illustrates their interdependence, with r/SuicideWatch consistently leading throughout, 2022 while the post count in r/Depression remained relatively stable.

### RQ2: How Do Users Transition From Participating in r/Depression to Engaging With r/SuicideWatch Subreddits?

The time-to-event algorithm was then used to analyze the data spanning from 2019 to 2022, encompassing the period before, during, and after the COVID-19 pandemic. The result showed that approximately 40% of users consistently took an average of 59 (SD 267) days to transition from their initial posts in r/Depression to their subsequent posts in r/SuicideWatch.

**Figure 2:**
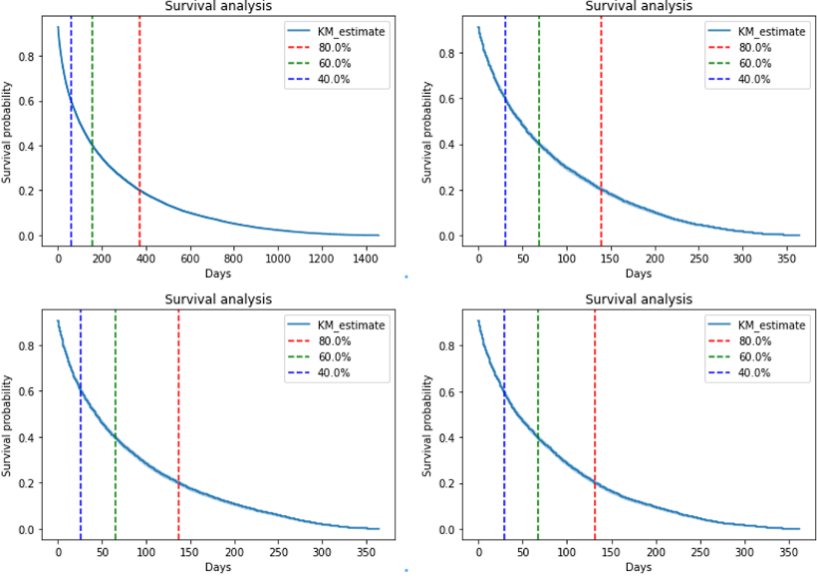
Time-to-event analysis.

To provide more detailed insights, we also applied the method for each separate year: 2019, 2020, and 2021 to 2022 (Figure 2). Distinct patterns emerged during this time frame. In 2019, 40% of users typically transitioned in about 30 days. However, in 2020, the median transition time notably decreased to 26 days, possibly influenced by the early challenges posed by the COVID-19 pandemic. Interestingly, in 2021 and 2022, we observed a slight increase in the median transition time, averaging around 29 days. It is worth noting that Figure 2 excludes data from 2022 due to its similarity to the 2021 pattern, highlighting the stability of this trend.

### RQ3: What Emotional Expressions and Signs of Depression are Prevalent Among Overlapping Users in r/Depression?

**Figure 3:**
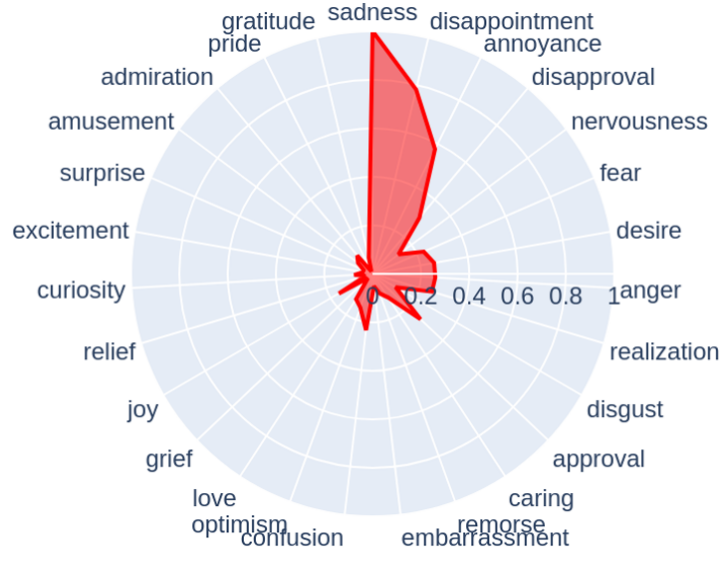
Emotional mean value.

Our analysis identified 26 emotions and calculated similarity values with the depression symptoms for each post. The radar chart in Figure 3 offers insights into the emotional content present in posts made by overlapping users in r/Depression subreddit. This group of users is likely to be at a higher risk of severe depression or SI. The chart indicates that sadness (1.00), disappointment (0.78), and annoyance (0.57) are the most frequently expressed emotions, aligning with the general mood and the difficulties typically associated with depressive states.

**Figure 4:**
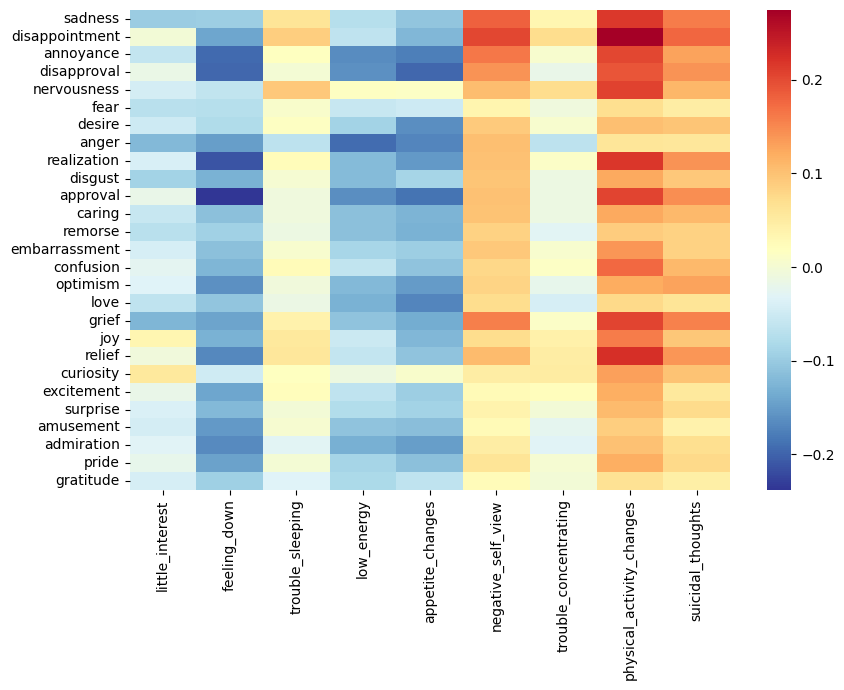
Correlation matrix of emotional and depression symptoms. PHQ-9: Patient Health Questionnaire–9.

The detailed distribution of depression symptoms and correlation matrix is provided in Multimedia Appendix 1. The similarity scores between social media posts and depression symptoms reveal that physical activity changes (0.72), negative self-view (0.70), and suicidal thoughts (0.69) are the symptoms most related in the analyzed content. Moreover, Figure 4 shows a strong correlation between suicidal thoughts and negative self-view, with a correlation score of 0.91, followed by physical activity changes with 0.84. Other notable symptoms, such as physical activity changes, show stronger relationships with trouble concentrating (0.79) and trouble sleeping (0.76). In addition, the sadness symptom has stronger positive relation with physical activity changes (0.22), negative self-view (0.18), and suicide thoughts (0.16).

### RQ4: What Topics Are Discussed by Overlapping Users in the r/Depression Subreddit, Categorized Based on SI Labels?

Through a series of iterative steps aimed at refining the accuracy of SI classification in Reddit posts, the initial fine-tuning of the classification model resulted in an accuracy of 0.62 for predicting SI labels for the original gold standard data set. Subsequently, the model’s accuracy improved from 0.62 to 0.67 after the first round, followed by a further improvement to 0.69, ultimately reaching 0.73 after 3 rounds of fine-tuning. The final model was trained using a learning rate of 4e−5, for 50 epochs, with a batch size of 64. In addition, a dropout layer with a parameter of 0.1 was incorporated into the model. This iterative methodology, coupled with the expansion of the gold standard data set and the meticulous data splitting and cross-validation, played a pivotal role in significantly enhancing the accuracy of SI classification in r/SuicideWatch posts. Consequently, our model demonstrated a substantial enhancement in its ability to identify potential instances of SI.

Within the r/SuicideWatch subreddit, posts were categorized based on users’ SI labeled using the Fine-Tuned DistilBERT model: a substantial 62% of the posted content was labeled as indicative of SI; in contrast, 38% of the content was categorized as not displaying suicidal thoughts. These statistics underscore a concerning presence of posts that revolve around suicidal thoughts and intentions within this subreddit. Our objective was to understand the different topics discussed by users in the r/Depression subreddit, who were categorized into “SI” and “non-SI” groups based on whether their posts in the r/SuicideWatch subreddit contain SI. As a result of the BERTopic modeling, Table 1 presents the top 10 topics for overlapping users’ posts in r/Depression, categorized by whether they have identified with SI in r/SuicideWatch. The complete topic information file is available in the GitHub link [39]**.**

**Table 1:** Top 10 topics from BERTopic model.

Topics			Posts allocated, n (%)
**Suicide ideation 1 (n=32,108)**			
	Struggling with thoughts of suicide and existential despair	5878 (18.31)	
	Struggles with depression and motivation in school and work	3639 (11.33)	
	Family relationship struggles and emotional trauma	2700 (8.41)	
	Sleep deprivation and mental exhaustion	2556 (7.96)	
	Relationship struggles and emotional turmoil	1908 (5.94)	
	Social anxiety and isolation	1843 (5.74)	
	Mental health struggles with medication and depression	1831 (5.7)	
	Difficulty in finding suitable therapy and dealing with therapist changes	1101 (3.43)	
	Coping with loss and supporting a friend in need	808 (2.52)	
	Substance use and mental health struggles	720 (2.24)	
**Suicide ideation 0 (n=6591)**			
	Mental health and suicidal thoughts	984 (14.93)	
	Family struggles and mental health woes	664 (10.07)	
	Feeling alone and unwanted in friendships	501 (7.6)	
	Struggling with depression and college decisions	493 (7.48)	
	Sleep struggles and relationship challenges	442 (6.71)	
	Dealing with unrequited love and friendship dynamics	334 (5.07)	
	Managing antidepressant medication and their effects	314 (4.76)	
	Understanding and coping with depression	294 (4.46)	
	Challenges and uncertainties in therapy and appointments	242 (3.67)	
	Relationship struggles and mental health crisis	210 (3.19)	

The BERTopic analysis revealed thematic clusters within posts of users labeled with SI. Users in this group actively participated in explicit discussions regarding suicidal thoughts, with a notable emphasis on the prevalence of topics centered on existential despair, which constituted 19% of the identified themes, followed by struggle with depression and motivation in school and work at 12%. In addition, social relationship struggle emerged across 4 topics, alongside difficulty and challenges related to depression medication and therapy. The heatmap matrix further explored the associations among the topics in this group. The most popular topic “suicide thought” showed a notable relationship (0.73) with “struggle with depression and motivations in school and work,” “sleep deprivation” (0.70), “mental health struggles during Covid lockdown” (0.73), and the theme around negative emotions and feelings. Furthermore, “struggle with depression and motivations in school and work” demonstrated stronger relations with topics such as “family relationship struggles” (0.74) and “substance use and mental health” (0.70).

Among users labeled *without SI*, notable topics include “mental health and suicidal thoughts” (984/6591, 14.93%), which is slightly less prominent than among users labeled with SI. In addition, discussions regarding family struggles and mental health constitute the second most popular discussed topic (664/6591, 10.07%). Topics related to relationship and feelings of isolation are prevalent among the rest of the topics. “Mental health and suicidal thoughts” also showed a higher relationship with topics such as “Understanding the impact of hate and criticism on people” (0.70). Moreover, the heatmap reveals a strong connection between the topics “family struggles and mental health woes” and “struggling with depression and college decisions,” with a similarity score of 0.73. Multimedia Appendix 1 provides a more comprehensive view of the topics.

## Discussion

The study used various NLP techniques and statistical methods to thoroughly explore user dynamics and behavior within the r/Depression community, particularly for those users who also engage in the r/SuicideWatch subreddit.

### Exploring Overlapping Users’ Behavior for RQ1 and RQ2

This study examined the behavior and dynamic of overlapping users within the 2 subreddits. Our result showed that 25,022 unique authors overlap between the communities, highlighting a shared user base. Notably, posts in r/SuicideWatch were shorter on average, with an average length of 373 words, compared with those in r/Depression, which had an average length of 767 words, shaping the nature of conversations. This contrasts with previous research that suggested a relationship between longer text lengths and users expressing SI on social media [40]. One possible explanation for this finding could be that individuals who participate in both communities may feel a greater need for profound emotion expression in the context of discussing depression stress or struggling in r/Depression community, leading to more succinct expression in the context of discussing suicidal thoughts within the r/SuicideWatch subreddit. Alternatively, it is conceivable that individuals who engage in overlapping discussions may have differing communication preferences or motivations for participation, leading to variations in post lengths between the 2 communities. Those longer posts may have a higher chance of being identified correctly due to their longer content [41].

A turning point occurred in August 2020, when the post count in r/SuicideWatch surpassed that of r/Depression, indicating evolving needs and interests. Before this shift, r/Depression had consistently held the lead in post count. However, after August 2020, their trajectories converged, demonstrating mutual influence. While the post count in r/Depression remained stable, albeit lower than that in 2019, post count in r/SuicideWatch maintained the lead in 2022. This potentially aligns with Centers for Disease Control and Prevention data indicating a record high in suicides in the United States in 2022 [42].

In addition, during the COVID-19 pandemic, suicidal behaviors showed an increase, and a sharp growing trend could be observed starting from June to August 2020 [43]. Our result revealed intriguing insights into the transition patterns of users from r/Depression to r/SuicideWatch. In 2019, users typically made the transition in approximately 30 days, reflecting a relatively consistent pattern. However, in 2020, there was a noticeable decrease in the median transition time to 26 days. This shift could be attributed to the unique challenges and stressors brought about by the early stages of the COVID-19 pandemic. Interestingly, in 2021 and 2022, we observed a slight increase in the median transition time, which averaged around 29 days. This suggests a potential adaptation or stabilization in user dynamics within these online communities as the pandemic persisted. These findings may be of interest from a public health perspective and for policy makers in navigating pandemic periods in the future.

### Analyzing r/Depression Posts by Overlapping Users in r/SuicideWatch for RQ3

The results imply that users are particularly concerned with changes in their negative self-view, physical activity, and SI, all of which are crucial indicators of more severe depression. A recent study [44] has shown that individuals with depression tend to view their present selves more negatively compared with their past or future selves, potentially exacerbating their depression. In addition, there was also a noteworthy correlation between changes in physical activity and trouble sleeping. This may be explained by the time spent indoor during the lockdown; however, less physical activity was directly associated with poorer sleep regularity, greater severity of insomnia, and greater depressive symptoms. Notably, during the lockdown, there was an indirect pathway from physical activity to depressive symptoms via insomnia severity [45].

The common stimulus (or information input) in suicide is intolerable psychological pain. Excruciating negative emotions, including shame, guilt, anger, fear, and sadness, frequently serve as the foundation for self-destructive behavior. These emotions may arise from any number of sources [46].

Research shows that comfort in expressing emotions, particularly sadness and happiness, is a protective factor against SI for young adults [47]. Sadness, disappointment, and annoyance are the most frequently expressed emotions that were identified in the posts of overlapping users in the r/Depression community. These findings suggest that suicide prevention efforts might have to be focused on increasing comfort in expressing emotions to mental health–related online communities as potential intervention targets.

Individuals with depression may experience a range of emotions, from gloom to grief, low self-esteem, and even pessimism, which may cause suicidal attempts or behaviors [48]. The heatmap data indicated strong positive correlations between the most frequent depression symptoms and certain negative emotional expressions, particularly disappointment. This multifaceted emotion often comprises frustration, anger, and sadness arising from uncertain expectations or desires [49]. It suggests that feeling of disappointment, prevalent among users’ posts, could be a key focal point for therapeutic interventions. Furthermore, emotions such as sadness, nervousness, and grief exhibited high correlation with these symptoms. While sadness and grief align with the typical depressive affect, their correlation with severe symptoms, such as suicidal thoughts, is particularly alarming and underscores the need for timely and effective support for individuals expressing these emotions. Our findings provide evidence that individuals with depression may endure prolonged feelings of sadness and a loss of interest in activities, alongside experiences of disappointment. If these emotions are not addressed appropriately, they may escalate to suicidal thoughts.

### BERTopic Analysis Combined With Fine-Tuned DistilBERT Classification Model for RQ4

By using a fine-tuned model on the r/SuicideWatch data set within the r/SuicideWatch subreddit revealed a concerning trend: 62% of the posts indicated SI, while 38% did not. This highlights a large volume of content related to suicidal thoughts and intentions within this online community. To delve deeper, we conducted an analysis at the user level, revealing that out of 163,993 unique users in the r/SuicideWatch subreddit, a staggering 72% engaged in discussions containing posts related to SI. The high prevalence of posts and user engagement focused on SI within the r/SuicideWatch subreddit is a clear sign of the substantial presence of individuals who are struggling with suicidal thoughts and may be in need of support and intervention.

The BERTopic analysis aimed to extract the hidden topical information from users’ posts in r/Depression subreddit, grouped by SI label. Not surprisingly, the largest theme prevalent in both groups is suicidal thoughts. Within the post of users labeled with SI, the second largest topic is struggle with depression and motivation in school and work. From the therapy prospect, lack of motivation, when depressed, can be a terrible problem [50]. In addition, these 2 topics also show higher correlation with family, relationship, substance use, and COVID-19 lockdown themes. This suggests the importance of considering the broader context in which depression and SI occur, including familial and social dynamics, and the impact of external stressors. Future research on SI may benefit from including a broader online community to generalize mental health issues related to SI.

In contrast, users labeled with no SI in the data set more commonly discuss mental health and family, followed by themes related to suicidal thought. Both groups discuss multiple topics related to family functioning and relationship struggles, as well as family relationship distress and depression, focusing on both children and adults within the family context [51]. Furthermore, antidepression topics related to the difficulty in finding suitable therapy and medicine in users with suicidal thoughts, challenges and uncertainties in therapy and physician appointments, antidepressant effects appear in posts by users without SI. Understanding the levels of therapy and medicine align with the mental health development processes for the 2 groups, enriching the efficiency of mental health interventions.

This is complemented by the understanding of lack of motivation, challenges in family relationships, barriers to obtaining therapeutic support, and the complexities of navigating intimate relationships. These factors may serve as key points in understanding depression, suicidal tendencies, and the pressing need for multifaced mental health support and intervention.

### Limitations

While this study provides insights into overlapping user behavior within the r/SuicideWatch and r/Depression Reddit communities, along with trends related to depression and SI during the pandemic, it is important to acknowledge the inherent limitations associated with this specific focus.

First, the study relies exclusively on data from the r/Depression and r/SuicideWatch Reddit communities. This limited data source may not fully capture the entire spectrum of individuals with mental health concerns, potentially leading to biased findings and limiting the generalizability of the results to other online platforms or offline population. While the study provides valuable insights, caution should be exercised when applying them beyond these specific communities. In addition, while the study used BERT models for classification and detection, it is important to note the model’s accuracy may not be optimal. This suggests that further refinement of the model may be required to improve its predictive accuracy. Given the observational nature of the study, it lacks the capacity to establish direct causal relationships between variables. While it effectively highlights user behaviors and trends, it does not delve into explaining the underlying reasons for the observed patterns.

In summary, while this study offers valuable insights into user behavior and trends within specific Reddit communities, these limitations should be considered when interpreting the findings and when planning future research in the field of online mental health support.

### Future Research

Expanding on the insights gained from this study, further research directions can address the identified limitations, providing a clear and more comprehensive understanding of online mental health support.

To mitigate the limitations tied to solely relying on Reddit communities, a pivotal step is diversifying data sources. Researchers in the future should consider incorporating data from a broader spectrum of online mental health platforms. In addition, future studies can delve into causal relationships among the identified topics or factors and conduct surveys, interviews, or experiments to uncover the factors influencing patterns in suicidal thoughts. The aggregation of data from various sources offers an opportunity to delve deeper into comprehending observed user behaviors, trends, and early signals related to mental health issues. Expanding the research scope beyond the specific Reddit communities to include various online mental health platforms, such as forums related to substance use, anxiety, addiction, and other mental health issues, can uncover distinct dynamics and themes. Furthermore, focusing on communities targeting specific age groups, such as teenagers or adults, could provide population-level analysis. Addressing these directions will contribute to a more holistic understanding of how individuals seek and receive support for their mental health concerns in digital spaces. These insights hold the potential to improve the effectiveness of online mental health interventions and support systems.

### Conclusions

Our research provides deeper insights into the dynamics of online mental health communities, particularly within the context of the COVID-19 pandemic and the increasing mental health challenges it has brought forth. The findings highlight the critical role that online platforms, such as Reddit, play in providing support and communication channels for individuals facing mental health difficulties, including thoughts of suicide.

The primary objective of this study was to gain a deeper understanding of the early signs of distress, specifically the transition from discussions centered on suicidal depression. The results revealed significant patterns and associations within the data, notably, the rapid transition from depression-related discussions to expressions of suicidal thoughts, particularly in 2020. Furthermore, the analysis of user posts in the r/Depression subreddit reveals a strong correlation between the expression of negative emotions, particularly disappointment, and the discussion of severe depressive symptoms such as changes in physical activity, negative self-view, and suicidal thoughts. Moreover, BERTopic analysis discovered different latent topics among the 2 groups within the overlapping user posts, with a prevalence of lack of motivation in the group with the SI label, while family struggle and mental health woes dominate in the group without SI label.

Overall, this study underscores the importance of online mental health communities in providing essential resources and support for individuals in need. The findings call for the development of early-stage online mental health support initiatives, along with an emphasis on fostering stable, responsive, and empathetic interactions within these platforms. Moreover, the integration of empathetic human chatbots and online community counseling could complement traditional methods of mental health support, offering a comprehensive approach to address the ongoing mental health challenges, including those exacerbated by the pandemic. This comprehensive approach holds the potential to bring about a meaningful and positive transformation in the realm of mental well-being, ensuring that individuals facing mental health difficulties receive the support they need in a timely and effective manner.

## Appendix

Multimedia Appendix 1 User dynamics and thematic exploration in r/Depression during the COVID-19 pandemic: insights from overlapping r/SuicideWatch users.

## Abbreviations

NLP: natural language processing
RQ: research question
SI: suicidal ideation

## Data Availability

The data sets generated during and analyzed during this study are available on the Internet Archive repository for “Reddit comments/submissions” [24].
