# Supplementary material for: User Dynamics and Thematic Exploration in r/Depression During the COVID-19 Pandemic: Insights From Overlapping r/SuicideWatch Users
Source: J Med Internet Res. 2024 May 20;26:e53968. doi: 10.2196/53968 (PMC11129781; doi:10.2196/53968)
Supplement: Multimedia Appendix 1 [file jmir_v26i1e53968_app1.docx]

# User Dynamics and Thematic Exploration in r/Depression during COVID-19: Insights from Overlapping r/SuicideWatch Users

# 1 Data and Methods

## PHQ-9 Depression *Symptoms* and associated sentences

Table 1 PHQ-9 Depression Symptoms and associated sentences

| **PHQ-9 Symptoms** | **Associated Sentences** |
| --- | --- |
| Little interest | "Little interest or pleasure in doing things" |
| Feeling down | "Feeling down, depressed, or hopeless" |
| Trouble sleeping | "Trouble falling or staying asleep, or sleeping too much" |
| Low energy | "Feeling tired or having little energy" |
| Appetite changes | "Poor appetite or overeating" |
| Negative self_view | "Feeling bad about yourself — or that you are a failure or have let yourself or your family down" |
| Trouble concentrating | "Trouble concentrating on things, such as reading the newspaper or watching television" |
| Physical activity changes | "Noticeable changes in movement or restlessness" |
| Suicidal thoughts | "Thoughts that you would be better off dead or of hurting yourself in some way" |

## *Model Explanation: Initial DistilBERT Model*

Our approach began by applied the DistilBERT model for suicide ideation (SI) classification using the gold standard dataset [33]. The methodology involved several key stages:

**Tokenization and Encoding:** Text data underwent preprocessing with the DistilBERT tokenizer, encompassing tokenization, truncation, and padding to ensure consistent input lengths. Training and test texts were encoded separately, resulting in numerical representations suitable for model training and evaluation.

**Model Definition:** The TFDistilBertForSequenceClassification model [32], a variant of the DistilBERT model pre-trained on a substantial text corpus, was employed. This model architecture has been tailored for sequence classification tasks, making it suitable for our SI classification. Initialized with 'distilbert-base-uncased' pre-trained weights, the model was configured to generate two labels: suicide ideation (SI) and No Suicide ideation(non-SI).

**Model Compilation:** Before training, the model was compiled using the Adam optimizer with a learning rate in the range of 3e-5 to 5e-5. The loss function was set to the model's compute_loss method, appropriate for sequence classification tasks. Accuracy was chosen as the evaluation metric to gauge the model's predictive performance.

**Model Training:** Model training involved shuffled training datasets batched into subsets of 16 samples. Training was performed over a range of 10-50 epochs, optimizing model weights based on calculated loss for each batch. The validation dataset provided ongoing performance monitoring during training.

**Prediction and Evaluation:** After training, the model was employed to predict SI labels for the test dataset. Predictions were generated using the model's predict method, producing logits for each sample. By applying the argmax function to the logit’s, predicted labels were obtained. Evaluation involved comparing predicted labels with the ground truth test dataset labels.

**Performance Metrics:** Model evaluation relied on accuracy as the primary metric, quantifying the proportion of correctly classified samples. Precision, recall, and F1 score could also be computed to provide a comprehensive assessment of the model's performance.

### BERTopic sub-models

Table 2: Tailored sub-models in BERTopic

| Sub-model Name | Parameter setting value | Description |
| --- | --- | --- |
| Embedding | “all-MiniLM-L6-v2” | This sub-model converts documents into number value, referred to as embedding. The all-MiniLM-L6-v2 model maps sentences and paragraphs to a 384-dimensional vector space for clustering or semantic search [35]. |
| Dimensionality reduction | UMAP (n_components=5, random_state=42, metric="cosine") | It reduces the dimensionality of document embeddings to create a more suitable format for us with HDBSCAN, resulting in effective clusters. |
| Clustering | HDBSCAN (metric='euclidean', prediction_data=True, metric-‘euclidean’) | After dimensionality reduction with UMAP, this sub-model clusters documents into similar groups. |
| Tokenizer | CountVectorizer( stop_words="english",nagram_range=(1,2)) | The sub-mode excludes English stopwords and extends the n-gram range to enhance topic representations consisting of one or two words. |

# Result

## Analyzing r/Depression Posts by overlapping users in r/SuicideWatch


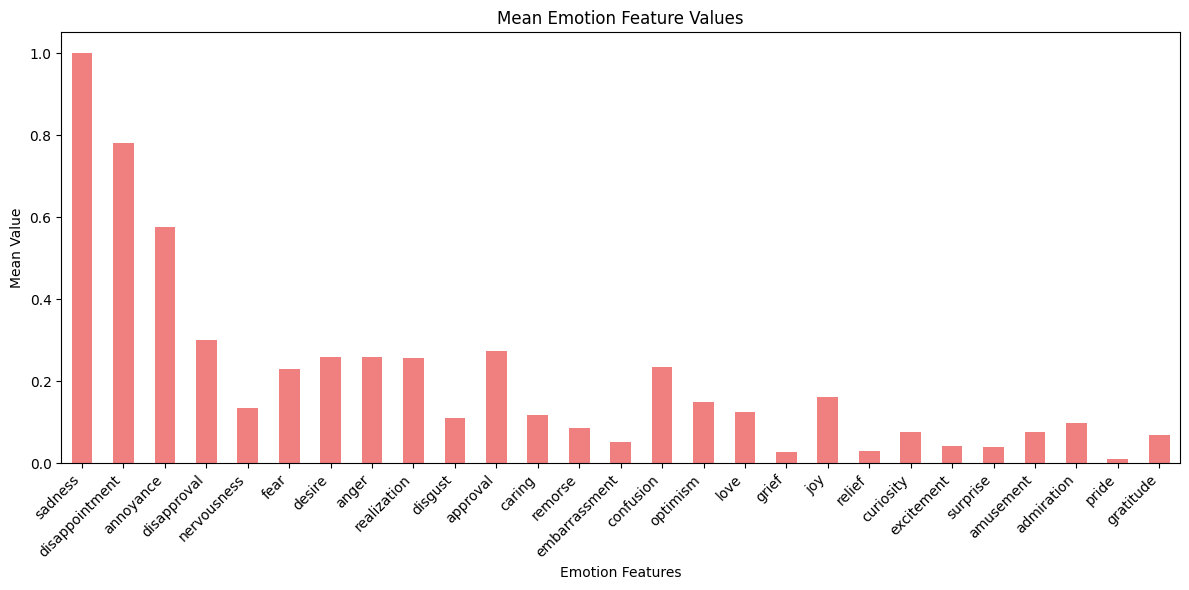


Figure 1 Distribution of mean Emotion value


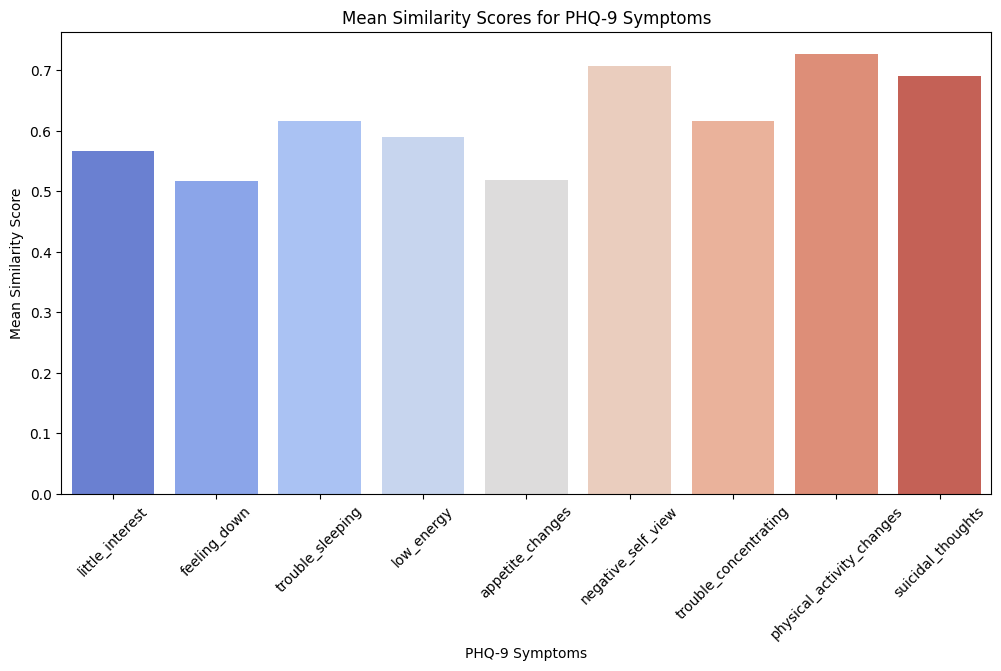


Figure 2 Distribution of mean similarity score between depression symptoms and posts


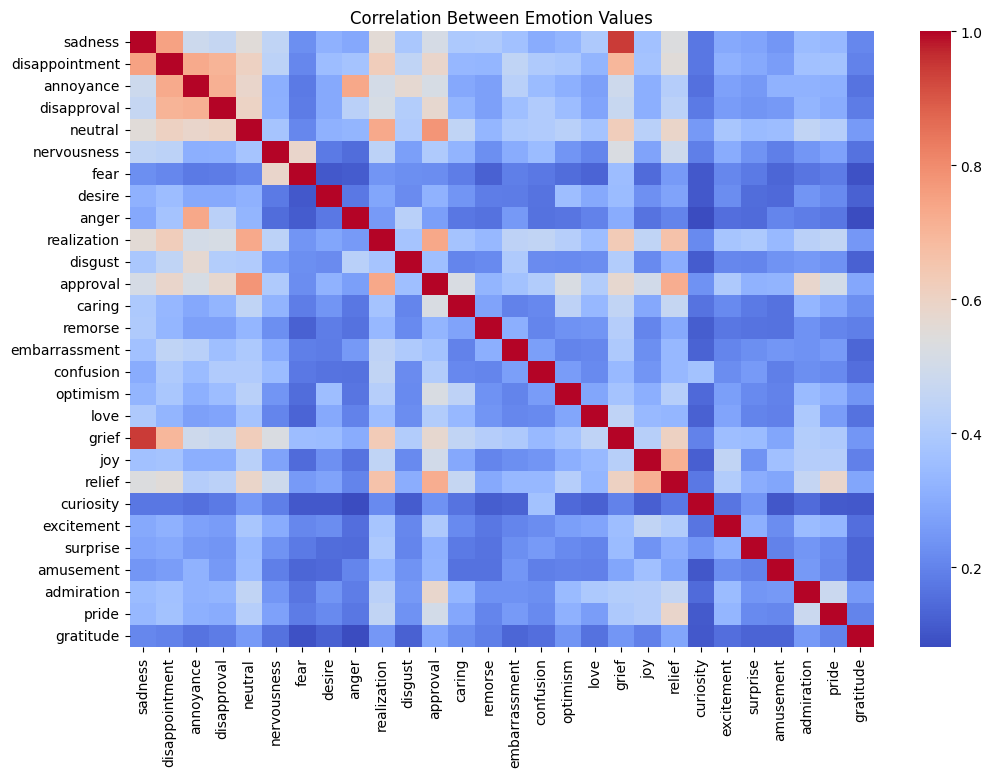


Figure 3 Correlation matrix between emotion value


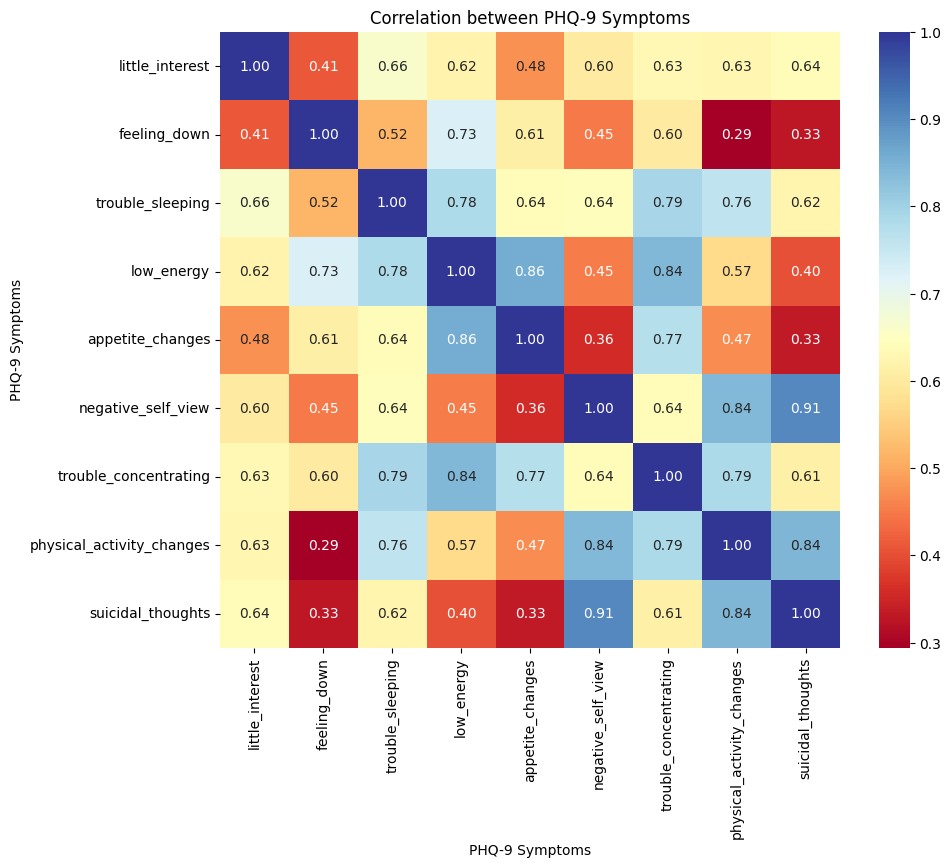


Figure 4 Correlation matrix between similarity score of depression symptoms

## BERTopic analysis Paired with Fine-Tuned DistilBERT Classification Model

As a result, the BERTopic [34] analysis revealed nine distinctive themes prevalent among posts from overlapping users who expressed suicidal ideation within the r/Depression subreddit. The following results represent the BERTopic results for the overlapping users labeled with suicide ideation in the r/Depression subreddit.

Table 3 Topics information for the overlapping users labeled with suicide ideation

| **Topic** | **Count** | **Name** | **Custom Name** |
| --- | --- | --- | --- |
| -1 | 37322 | -1_just_im_like_feel | Mental health struggles and emotional turmoil |
| 0 | 5878 | 0_life_want_die_wish | struggling with thoughts of suicide and existential despair |
| 1 | 3639 | 1_job_im_just_school | Struggles with Depression and Motivation in School and Work |
| 2 | 2700 | 2_mom_dad_just_im | Family Relationship Struggles and Emotional Trauma |
| 3 | 2556 | 3_sleep_wake_bed_day | Sleep Deprivation and Mental Exhaustion |
| 4 | 1908 | 4_just_like_friend_know | Relationship struggles and emotional turmoil |
| 5 | 1843 | 5_friends_friend_people_just | Social Anxiety and Isolation |
| 6 | 1831 | 6_mg_meds_taking_feel | Mental Health Struggles with Medication and Depression |
| 7 | 1101 | 7_therapist_therapy_help_just | Difficulty in Finding Suitable Therapy and Dealing with Therapist Changes |
| 8 | 808 | 8_hes_just_friend_know | Coping with loss and supporting a friend in need |
| 9 | 720 | 9_alcohol_weed_drink_drunk | Substance Use and Mental Health Struggles |
| 10 | 656 | 10_cut_harm_self harm_self | Self-harm struggles and seeking support |
| 11 | 642 | 11_look_ugly_hair_like | Struggling with self-image and body acceptance |
| 12 | 602 | 12_talk_someon_need_someon talk | Need someone to talk, feeling lonely and awful |
| 13 | 587 | 13_eat_eating_food_weight | Eating habits and mental health |
| 14 | 425 | 14_games_game_play_video | Struggles with Video Game Addiction and Loss of Interest |
| 15 | 349 | 15_cri_crying_tears_feel | Coping with overwhelming emotions through crying |
| 16 | 346 | 16_teeth_shower_clean_room | Personal Struggles with Dental Hygiene, Room Cleanliness, and Self-Care |
| 17 | 345 | 17_depress_depression_depressed_like | Understanding Depression and Supporting Those Who Feel Depressed |
| 18 | 335 | 18_numb_feel_like_emot | Coping with Numbness and Emptiness in Emotional State |
| 19 | 317 | 19_post_posts_social media_media | Social Media Support Group Guidelines |
| 20 | 313 | 20_birthday_year_today_just | feelings of sadness and loneliness on birthdays |
| 21 | 312 | 21_dog_cat_cats_just | Struggles and Emotions of Pet Owners |
| 22 | 294 | 22_sorry_im sorry_love_poem | Apologies and self-reflection through poetry about love, pain, and struggles |
| 23 | 293 | 23_im_gay_just_like | Struggles with Sexual Orientation and Depression |
| 24 | 253 | 24_hug_want hug_want_hugged | Needing Real Hugs |
| 25 | 246 | 25_covid_lockdown_virus_quarantine | Mental Health Struggles During Covid Lockdown |
| 26 | 234 | 26_happi_happy_happiness_feel | Understanding Happiness and Overcoming Sadness |
| 27 | 231 | 27_music_listen_song_songs | Loss of Enjoyment in Music and Coping Strategies |
| 28 | 226 | 28_care_cares_peopl_cares cares | Mental Health and Feeling of Neglect - Exploring Themes of Care, Support, and Desperation |
| 29 | 204 | 29_motiv_motivation_just_work | struggles with motivation and depression |


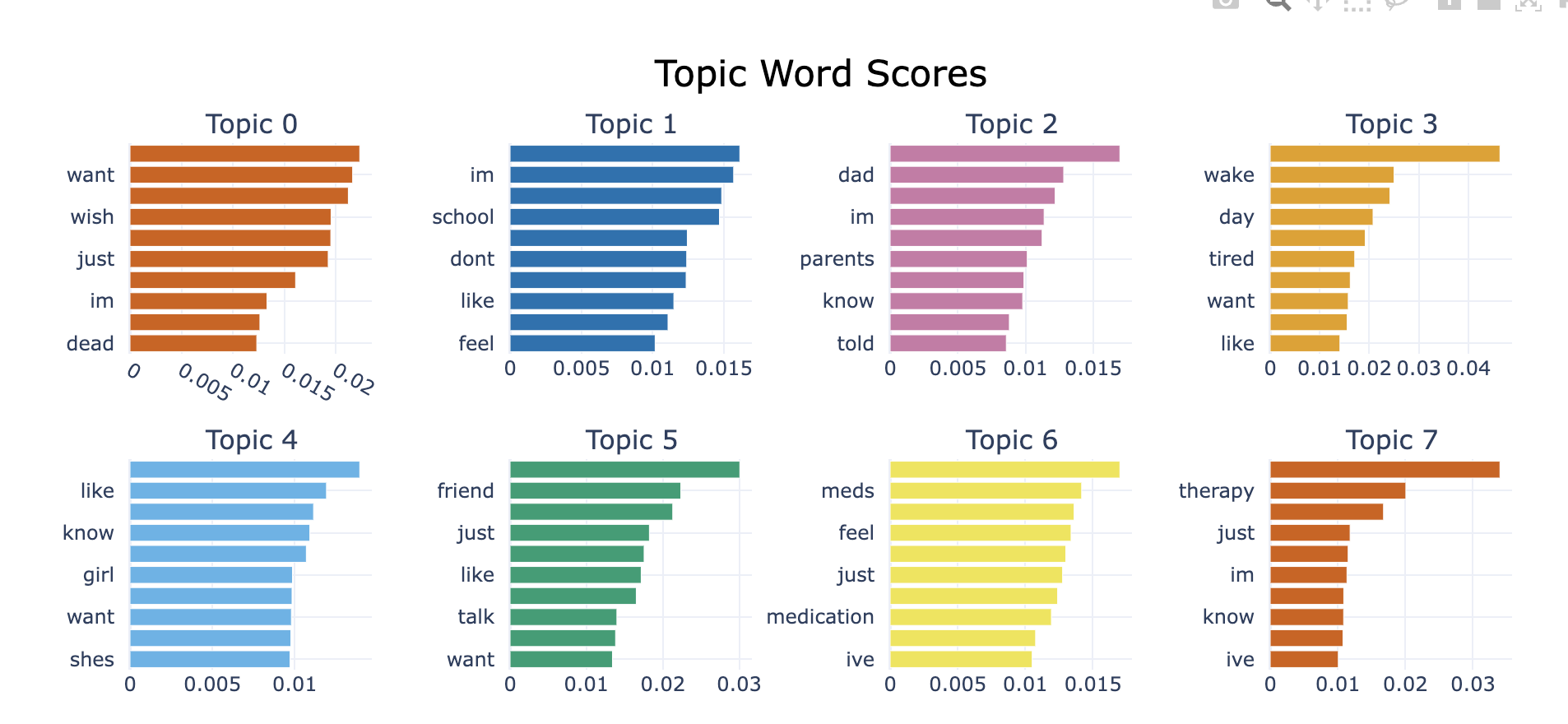


Figure 5 Word scores of top ten topics


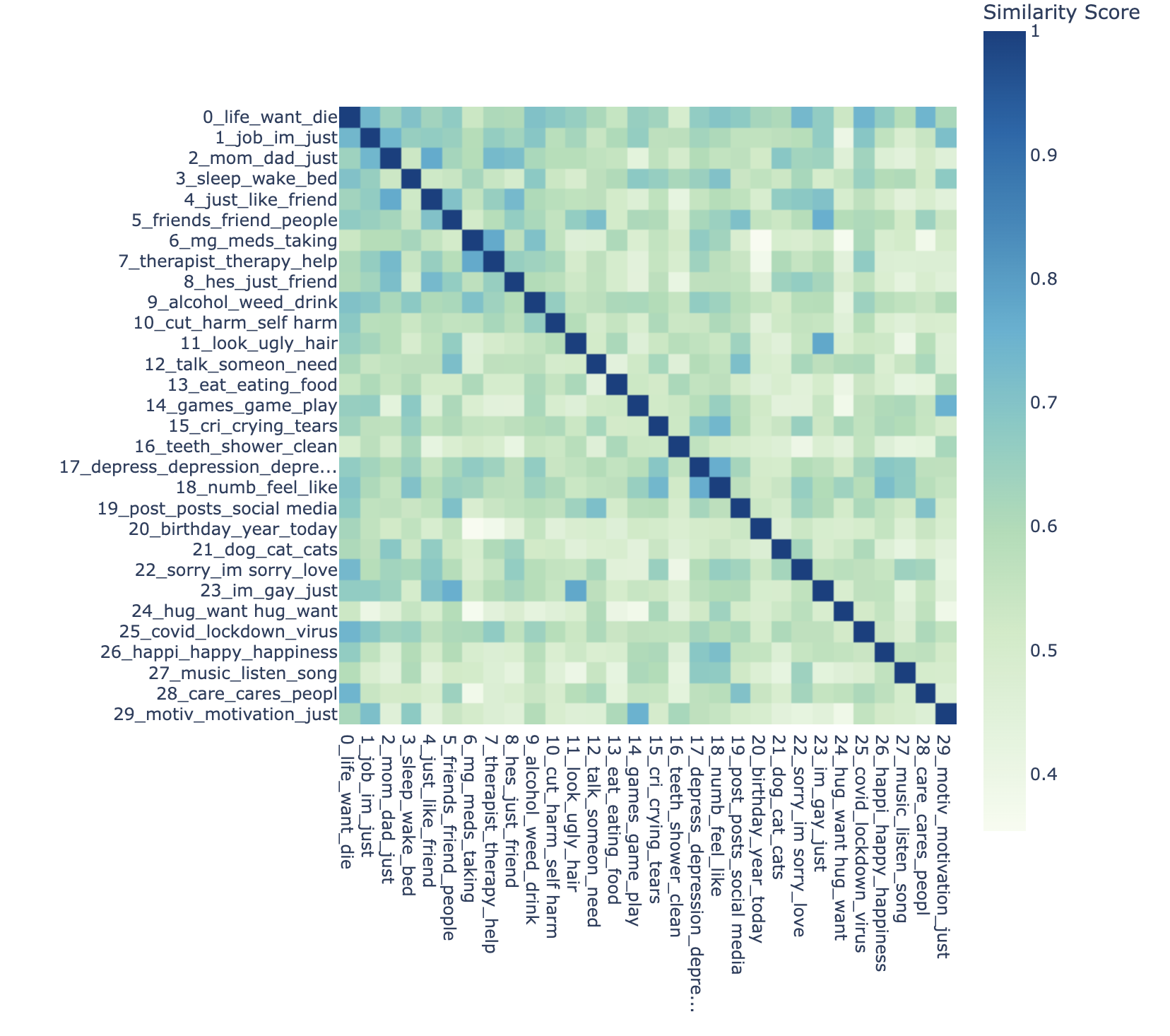


Figure 6 Correlation matrix of topics

The following table and figure represent the BERTopic results for overlapping users’ post without suicide ideation in r/Depression subreddit.

Table 4 Topics information for the overlapping users labeled without suicide ideation

| **Topic** | **Count** | **Name** | **Custom Name** |
| --- | --- | --- | --- |
| -1 | 8180 | -1_just_im_like_feel | Mental Health Struggles and Suicidal Thoughts in Difficult Circumstances |
| 0 | 984 | 0_die_die want_want die_want | mental health and suicidal thoughts |
| 1 | 664 | 1_mom_just_im_dad | Family Struggles and Mental Health Woes |
| 2 | 501 | 2_friends_friend_people_feel | Feeling Alone and Unwanted in Friendships |
| 3 | 493 | 3_job_school_im_just | struggling with depression and college decisions |
| 4 | 442 | 4_sleep_day_wake_bed | Sleep Struggles and Relationship Challenges |
| 5 | 334 | 5_friend_just_know_like | Dealing with unrequited love and friendship dynamics |
| 6 | 314 | 6_mg_taking_meds_prozac | Managing antidepressant medication and their effects |
| 7 | 294 | 7_depress_depression_better_feel | Understanding and Coping with Depression |
| 8 | 242 | 8_therapist_therapy_help_im | Challenges and uncertainties in therapy and appointments |
| 9 | 210 | 9_friend_just_know_like | Relationship Struggles and Mental Health Crisis |
| 10 | 176 | 10_talk_someon_need_someon talk | Need to Chat with Someone |
| 11 | 176 | 11_motiv_like_just_feel | Loss of Motivation and Joy in Daily Activities |
| 12 | 162 | 12_ugly_look_im_ugli | Seeking help for self-image and appearance struggles |
| 13 | 157 | 13_cut_self_harm_self harm | Self-Harm Reduction Strategies |
| 14 | 116 | 14_drunk_drink_alcohol_drinking | Coping with depression and thoughts of suicide through alcohol, substance abuse, and unhealthy behaviors |
| 15 | 105 | 15_care_peopl_people_cares | Lack of Genuine Care and Support |
| 16 | 104 | 16_cri_crying_cried_just | Crying as Catharsis and Taboo in Society |
| 17 | 101 | 17_eat_food_eating_weight | Eating Habits and Weight Struggles |
| 18 | 98 | 18_teeth_room_clean_shower | Struggling with maintaining personal hygiene and room cleanliness |
| 19 | 80 | 19_numb_feel_feeling_pain | Coping with Numbness in Depression |
| 20 | 79 | 20_dog_cat_pet_cats | Struggles with Pet Ownership and Emotional Support |
| 21 | 69 | 21_hate_people_hate hate_hatred | Understanding the Impact of Hate and Criticism on People |


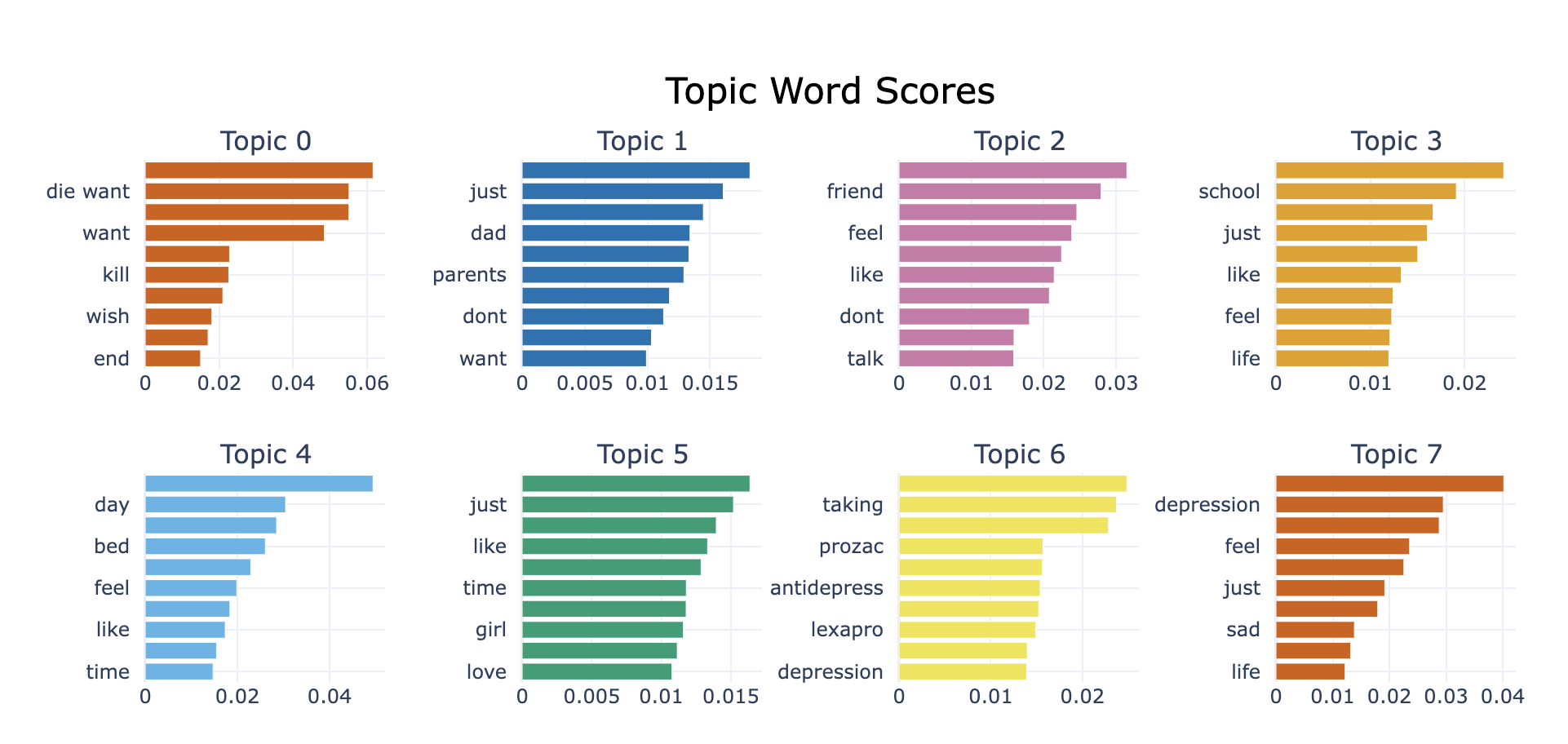
 Figure 7 Word scores of top ten topics


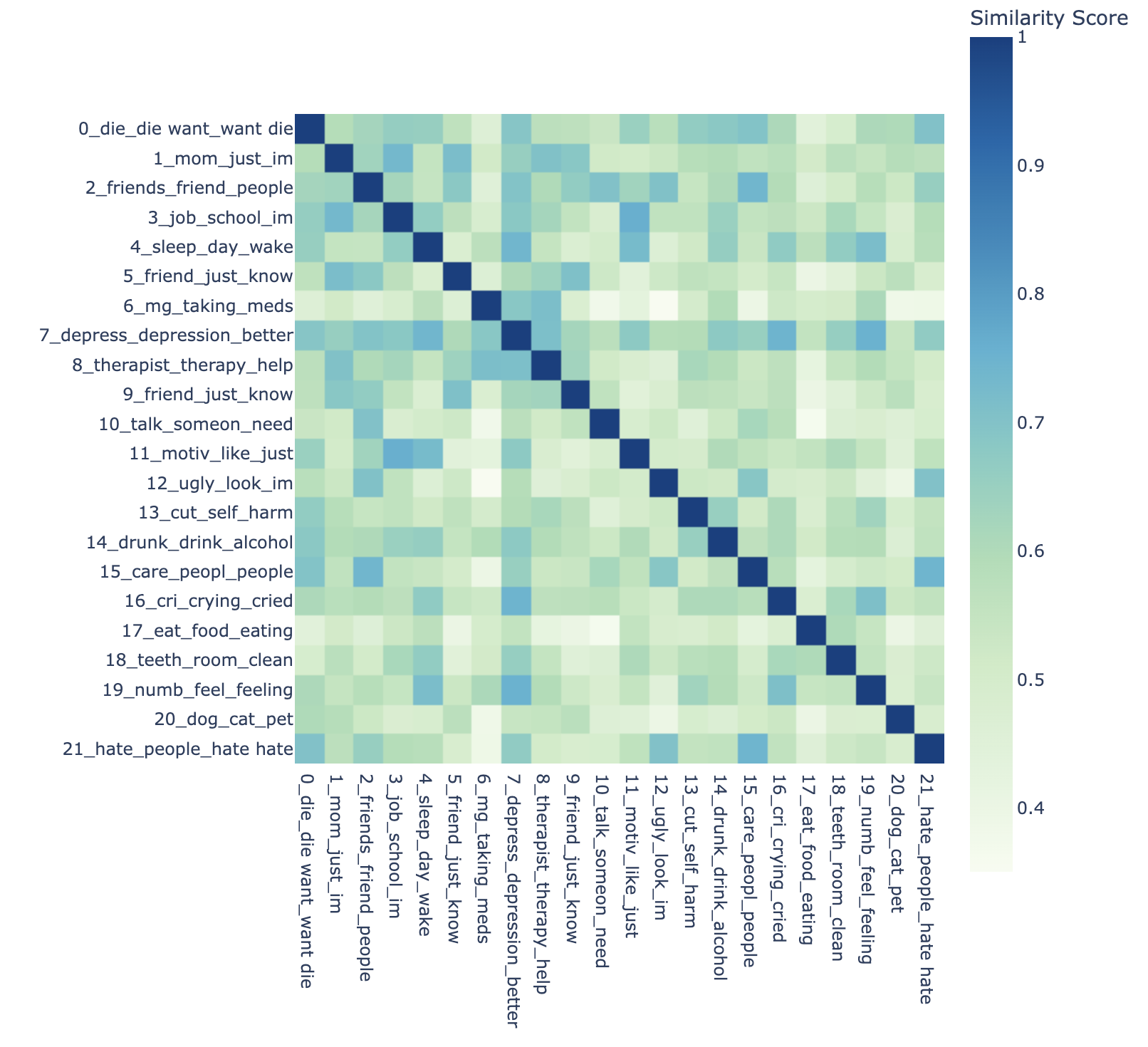


Figure 8 Correlation matrix of topics
